# Investigation of membranes-electrodes assemblies in anion exchange membrane fuel cells (AEMFCs): Influence of ionomer ratio in catalyst layers

**DOI:** 10.1016/j.heliyon.2024.e29622

**Published:** 2024-04-12

**Authors:** Zarina Turtayeva, Feina Xu, Jérôme Dillet, Kévin Mozet, Régis Peignier, Alain Celzard, Gaël Maranzana

**Affiliations:** a Université de Lorraine, CNRS, LEMTA, F-54000, Nancy, France; b Université de Lorraine, CNRS, IJL, 88000, Épinal, France; c Electrochemistry Laboratory, Paul Scherrer Institut, PSI, 5232, Villigen, Switzerland

**Keywords:** AEMFCs (Anion exchange membrane fuel cells), Catalyst inks, CCMs (Catalyst-coated membranes), GDEs (Gas-diffusion electrodes), Polarization curves, Water management

## Abstract

Anion exchange membrane fuel cells (AEMFCs) have recently attracted significant attention as low-cost alternative fuel cells to traditional proton exchange membrane fuel cells because of the possible use of platinum-group metal-free electrocatalysts. Over the past decade, new materials dedicated to the alkaline medium, such as anion exchange membranes (AEMs) and anion exchange ionomers (AEIs), have been developed and studied in AEMFCs. However, only a few AEMs and AEIs are commercially available, and there are no ready-to-use membrane electrodes assemblies (MEAs) with the desired AEMs and AEIs. Consequently, the need to manufacture in-house CCMs or GDEs becomes a reality that we must face.

This work deals with the influence of ionomer content on the prepared MEAs with the commercial anion exchange membrane and ionomer from Aemion™ Ionomr Innovations AF1–HNN8–2 and AP1–ENN8/HNN8 respectively and by varying the support (gas diffusion layer or membrane). The prepared MEAs were characterized morphologically by SEM and profilometry, as well as electrochemically by AEMFC polarization curves and cyclic voltammetry. In addition, an attempt to investigate water management was made with and without a reference electrode in the cell to understand the behavior of water in an operating AEMFC. Our results show that CCM-based MEAs can undergo deformation during the anion conversion step, leading to weakening of the membrane and hence faster degradation in the fuel cell. On the contrary, no deformation was observed for the GDEs during the anionic conversion, although the results are poorer due to (i) poor interface contact between membrane and GDE that depends on ionomer ratio in the ink and (ii) a high overpotential at the anode due to the production of water that cannot be effectively evacuated.

## Highlights

• The optimum ink composition is different for GDE – MEAs and CCM – MEAs.
• Higher the ionomer content – lower the OCV of CCM – MEAs.
• The higher the amount of ionomer, the greater the segregation in the electrode.
• The thickness of the Teflon gasket can stabilize water management.

## 1 Introduction

AEMFC is an electrochemical device which can convert H_2_ and O_2_ to water and electricity via an anion exchange membrane (AEM). Contrary to the mature PEMFC, the development of AEMFC seems to be boosted during the last decade. This surge of interest in AEMFC can be explained by its potential low cost due to the possibility of using non-noble catalysts for the oxygen reduction reaction (which can be more available and cheaper than Platinum Groupe Metal (PGM) catalysts), non-perfluorosulfonated membranes and by the favorable kinetic of the ORR (oxygen reduction reaction) in alkaline media [[1], [2], [3]].

Although substantial progress has been achieved over the last decade in the development of anion exchange membranes (AEMs) and anion exchange ionomers (AEIs), such as higher OH^−^ conductivity and improved durability [[4], [5], [6], [7], [8], [9]], only a few of AEMs and AEIs are commercially available. Among them, Aemion™ membranes from Ionomr Innovations can be used for AEMFC applications [[10], [11], [12]], where a current density of 1.5 A cm^−2^ was reported in the literature at 0.3 V for example. However, this result cannot be attributed solely to the membrane feature, but to the know-how the authors possess in bringing together all the elements of a MEA and making them work properly in an AEMFC.

A MEA is composed of a membrane sandwiched between two catalyst layers and two gas diffusion layers (GDLs), and there are two approaches to MEA fabrication. The first is the combination of a catalyst layer and a gas diffusion layer, which forms the gas diffusion electrode (GDE) or catalyst coated backing (CCB) and is placed on both sides of a membrane [[13], [14], [15]]. The second is the combination of two catalyst layers and a membrane, which forms the catalyst-coated membrane (CCM) [10,16,17]. Then, the CCM is clamped between two GDLs. Unfortunately, there are only a few articles dealing with MEA fabrication for AEMFCs [5,[18], [19], [20], [21]], because of the recent development of alkaline fuel cells and new dedicated materials such as AEMs and AEIs. That is why, there is still a need for further research to develop effective and scalable MEA fabrication methods which can be used in AEMFCs.

Of all the elements that compose a MEA, the catalyst layer (CL) is one that cannot be bought off the shelf. CL is generally obtained from a catalyst ink which is usually composed of a catalyst (supported or not on carbon), ionomer and solvent(s). Its formulation and preparation can vary according to the ingredients chosen. For instance, Mustain et al. [22] reported two types of MEAs fabrication methods that have shown high MEA performance and durability: (i) Los Alamos standard method with ionomer binder [23,24] and (ii) USC/Surrey method with ionomer powders [7,19]. The Los Alamos standard method prepares an ionic dispersion binder in hydroxide form, before preparing an ink, claiming that halogen counter anions (fluoride (F^−^), chloride (Cl^−^), bromide (Br^−^), iodide (I^−^) and astatide (At^−^)) should be avoided, as they severely poison catalysts. In contrast, USC/Surrey method prepares ink without prior anionic exchange of ionomer in powder form.

As far as solvents are concerned, they are considered sacrificial substances because they are usually evaporated during coating to obtain a solid catalyst layer. However, they are necessary to dissolve the ionomers and disperse the catalyst and supported carbon nanoparticles. Depending on the solvent's properties and their interaction with ionomers and solid particles (catalysts and carbon), the resulting ink has its own properties in terms of dispersion, viscosity, solubility, or hydrophilicity/hydrophobicity. Consequently, the morphology of the obtained CL and its performance may also be affected by the quality of the prepared ink. According to the literature [13,[25], [26], [27]], optimization of the performance of CLs was performed by varying solvents when prepared catalyst inks for a given ionomer.

To optimize the ink formulation and the performance of the AEMFC, it is important to adjust the amount of ionomer because the type of ionomer can affect the morphology of the resulting electrodes, and consequently the mass transport. But there are not many works dealing with this topic in the literature [25,[28], [29], [30]]. Among the works cited, Hernandez-Flores et al. [30] studied the effect of the concentration of ionomeric solution on the hydrogen oxidation reaction (HOR) and ORR by rotating disc electrode (RDE). It is important to note that although the kinetic of the HOR in alkaline media is lower than in acidic media, the ORR still remains the limiting reaction in alkaline media by 2-3 orders of magnitude [31]. Under such RDE operating conditions, using liquid KOH as electrolyte, the authors [25] showed that the kinetic and mechanism of the reactions are affected by the ionomer loading in the catalyst layer. However, it is a pity that the type of ionomer solution was not mentioned in the article for the understanding of the results, especially in the case of 0.1 % of ionomer. While, using commercial ionomer and membrane, such as Fumion and FAA-3-20, respectively, Sebastian et al. [29] pointed out that 25 % of the ionomer ratio gives the poorest performance in AEMFC whereas the optimal loading was achieved with 50 % of the ionomer. This value is high compared with literature but could be explained by the unconventional method of the anionic conversion step of the membrane and electrodes, which takes place in a mixture of water/isopropanol solution (75/25 %). Another work with a commercial ionomer and membrane from Tokuyama Corp. (AS-4 solution and A-201 membrane) was reported by Yang et al. [25], who prepared various catalyst inks, in particular with different amount of ionomer and isopropanol as solvent. They demonstrated that 20 % ionomer loading is the optimum to achieve the best performance in the AEMFC. Furthermore, they studied the pore structure of the obtained CCM by the MIP method (mercury intrusion porosimetry) for explaining the results in AEMFC. The microstructure data of the obtained CCMs at different ionomer loading showed that (i) the cumulative pore volumes of the CCMs decreases sharply with the increase of ionomer content and (ii) the mean pore volume diameter appears to increase with increasing ionomer content, except for the 20 % wt. ionomer sample. Unlike mature PEMFCs, where membranes and perfluorosulfonated ionomers are widely used, there is a wide variety of AEMs and AEIs that can be used in AEMFCs. Therefore, all the works reported here was performed with different types of ionomers and membranes.

Although many research groups through the world have attempted to fabricate CLs and MEAs for AEMFCs [13,25,26,32,33], it should be noted that most of the highly performing MEAs have been obtained from non-purchasable materials. However, the study of AEMFC in a fundamental aspect, for instance, the water management issue and the relationship between CLs structure and triple phase boundary, requires the development of this type of fuel cell on a laboratory scale, in particular with commercial materials. In fact, water management is more severe in AEMFCs because the ORR in alkaline media requires water molecules and twice as much water is produced on the anode side as in PEMFCs (2H_2_ + 4OH^−^ → 4H_2_O + 4e^−^ vs O_2_ + 4H^+^ + 4 e^−^ → 2H_2_O respectively in AEMFC (anode) and PEMFC (cathode)). As shown in literature [3,34,35], there are different strategies to study the water management issue in an AEMFC, such as the type of gas diffusion layers (GDLs), the relative humidity of gas or the inlet flow for example. Before varying the operating conditions, a MEA is required with an AEM and working electrodes in alkaline media. Since there are only a few commercially available AEMs and AEIs and no ready to use MEAs with the wished AEMs or AEIs, it is necessary to manufacture CCMs and/or GDEs based on commercial materials. For this, catalyst ink coating steps need to be mastered before testing CCMs and GDEs in AEMFC.

In this work, the commercial anion exchange membrane and ionomer from Aemion™ Ionomr Innovations AF1–HNN8–2 and AP1–ENN8/HNN8 respectively were chosen to fabricate CCM and CCB-based MEAs. This work attempts to establish a relationship between the morphology of prepared MEAs and their electrochemical behavior in AEMFC. Indeed, the way of building a MEA plays a critical role in the behavior of polarization curves in an AEMFC, due to the modification of the CL structure and the type of support chosen. However, the optimal design of catalyst layer structures is yet to be explored despite its importance. Due to our lack of knowledge about anionic materials and testing, a systemic study was performed with in-house CCMs and GDEs, by varying ionomer content from 13 to 33 % in weight within catalyst inks. Additionally, an attempt was made to investigate water management with a reference electrode in the cell to understand the behavior of water in an operating AEMFC. The prepared samples were characterized morphologically by SEM and profilometer techniques and electrochemically by AEMFC polarization curves.

## 2 Experimental part

### 2.1 Materials

The AP1–ENN8/HNN8 soluble ionomer powder and AF1–HNN8–25 membrane sheets were purchased from Aemion™ Ionomr Innovations. 40 wt % Pt supported on carbon 10.13039/100007274Vulcan XC – 72R and Sigracet® GDLs 28BCE with a microporous layer and PTFE (5 wt %) were purchased from Fuel Cell Store. Isopropanol (IPA) with 99.8 % purity was purchased from Carlo Erba. Methanol with 99.9 % purity was purchased from Sigma-Aldrich. High-purity demineralized water (18 MΩ cm) was used in this work.

UltraTurrax, a high shear disperser (IKA® T 10) was used in the preparation of catalyst inks.

All coating layers were obtained with an automatic ultrasonic spray bench (ExactaCoat) from SonoTek. An ACCUMIST nozzle (120 kHz) was used in this work. A 1 mm thick polycarbonate (PC) mask was used during coating.

A Dektak XT tactile profilometer (Bruker) was used to measure the thickness of the deposited layers. The probe size of the profilometer was 2 μm and the measurement error was approximately 10 nm. The measurement step varied depending on the time required to perform the measurement. The maximum sample length that the device could measure was 4 cm.

The morphology of the deposited layer was visualized using the SEM technique (Gemini SEM 500 from Zeiss).

### 2.2 Catalyst inks preparations

First, a 5 wt% ionomer stock solution was prepared by dissolving AP1–ENN8/HNN8 ionomer as a powder in methanol [10,36]. The catalyst inks were prepared with 0.4 g of Pt/C, varying the ionomer content from 3 to 33 %, with a 10 % step. The ionomer content (R(ionomer)) was defined as the weight of the total solid content of the catalyst inks, as expressed in Eq. (1).

(1) $$R\left(ionomer\right)=\frac{m\left(solid\;ionomer\right)}{m\left(solid\;ionomer\right)+m\left(catalyst\;powder\right)}$$

where m(solidionomer) is the mass of API-ENN8/HNN8® ionomer in solid form and m(catalystpowder) is the mass of catalyst powder in the dispersion.

Four inks containing different amounts of ionomer (3, 13, 23 and 33 %) were prepared with the dispersion step. A mixture of water and isopropanol in a ratio of 1:9 was used for all prepared inks. The general description of the ink preparation with the dispersion step can be described as follows. First, 0.4 g of Pt/C was introduced in a bottle of 100 ml. Then, distilled water was poured over the catalyst powder to avoid ignition of the platinum nanoparticles upon direct encounter with isopropanol (IPA). Afterward, a half of IPA amount was added to the catalyst and water mixture. The obtained dispersion was agitated for 30 min with magnetic stirring and followed by a high shear dispenser step around 16,000 rpm during also 30 min. After this homogenization step, the ionomer solution and the rest of IPA were added. The final catalyst ink was agitated with magnetic stirring overnight. In addition, all ink preparation steps were carried out at room temperature.

### 2.3 MEAs based on CCM and GDE

#### 2.3.1 Manufacturing of CCMs and GDEs

Once the described catalyst inks were prepared, they were fed separately into the ultrasonic spray coating bench to fabricate CCMs and GDEs samples, with the membrane AF1–HNN8–25 and GDL 28 BCE as supports, respectively. The spray coating was identical to that described in Ref. [37], *i.e.*, using an ultrasonic spray bench (ExactaCoat, from SonoTek), at a nozzle height of 60 mm, with a spray pattern consisting of 2 vertical and 2 horizontal serpentine layers (configuration C mentioned in Ref. [37]) and a 1 mm thick polycarbonate mask. The membrane or the GDL was placed on a heating table set at 80 °C. The latter is the maximum membrane operating temperature specified by the supplier and also used in the literature [12]. Unless specified, the Pt loading of the prepared MEAs is set at 1 mg cm^−2^ for both electrodes with an active area of 7.22 cm^2^.

For convenience, we call the CCMs and GDEs obtained with the four inks prepared with the dispersion step, containing different amounts of ionomer (X%) and a Pt loading of 1 mg cm^−2^ as such: CCM-X% and GDE-X%, respectively. Thus, we must deal with at least 8 types of samples: CCM-3%, CCM-13 %, CCM-23 %, CCM-33 %, GDE-3%, GDE-13 %, GDE-23 % and GDE-33 %. In the case of a lower Pt loading such as 0.5 mg cm^−2^ (see below), the sample name will be specified in the text as GDE-23%-0.5 (Table 1).

**Table 1:** Composition and preparation of the catalyst inks.

Ionomer (wt. %)	Preparation mode	Pt loading (mg.cm^−2^)	MEA
3	mixed	1	CCM-3% GDE-3%
13	mixed	1	CCM-13 % GDE-13 %
23	mixed	1	CCM-23 % GDE-23 %
		0.5	GDE-23%-0.5
	non – mixed^a^		GDE-23%-unmixed
33	mixed	1	CCM-33 % GDE-33 %

a The non-mixed mode is ink preparation without the use of the ultra-Turrax step.

#### 2.3.2 Characterization of catalyst layers (CLs)

Special samples dedicated to SEM images and profilometry were prepared using the 4 inks described. The morphology of the CLs was observed by SEM, and the thickness and homogeneity of the CLs were carried out by profilometry. The porosity of the CLs obtained was estimated using Eq. (2).

(2) $$Porosity=\frac{\left(S_{A}\times t_{CL}-R\left(ionomer\right)\times \frac{m_{CL}}{\rho_{ionomer}}-\left(1-R\left(ionomer\right)\right)\times \frac{m_{CL}}{\rho_{Pt/C}}\right)}{S_{A}\times t_{CL}}$$

where S_A_ the surface area of the electrode (7.22 cm^2^), t_CL_ is the thickness of catalyst layer (CL) measured by the profilometer, R(ionomer) is calculated from Eq (1), m_CL_ is the deposited mass, ρ_ionomer_ is the density of Aemion ionomer at 1.2 g cm^−3^ and ρ_Pt/C_ is the density of Pt/C 40 % wt powder, estimated to 8.64 g cm^−3^ (with 0.096 and 21.45 g cm^−3^ for the carbon Vulcan XC-72R and Pt, respectively).

#### 2.3.3 General procedure of MEAs fabrication

All the prepared CCMs and GDEs were stored at room temperature, before being used in the membrane electrode assemblies. Before MEAs were manufactured, ionic conversion was mandatory to exchange the iodide to hydroxide anions. For that, all samples were soaked in 3 M KOH (high purity) for 48 h (protocol given by the supplier), with a bath change every 12 h, and washed with a large amount of demineralized water. It is worth to notice that the Aemion® membrane was received in the form of iodide (a form of counter-anion), which is difficult to remove without a highly concentrated KOH solution. In fact, other anion conversion methods (such as using a one-step conversion with 1 M KOH or a two-step conversion with 1 M NaCl then 1 M KOH) were tested and compared to remove the iodide anion, but the method used gave us the highest ionic conductivity with the lowest membrane volume deformation (V_wet_/V_dry_ = 2). Visually, no degradation such as membrane dissolution or embrittlement in 3 M KOH was observed during the anion conversion step.

Unless otherwise specified, CCM-based MEAs were composed of two 28 BCE GDLs, two PTFE gaskets (100 μm), two PET sub-gaskets (25 μm) and an in-house CCM in OH^−^ form for a given catalyst ink. GDE-based MEAs were composed of two in-house GDEs in OH^−^ form for a given catalyst ink, two PTFE gaskets (100 μm), two PET sub-gaskets (25 μm) and a piece of the Aemion® membrane in OH^−^ form. No hot-press was used during the assembly of the CCMs and GDEs. Regardless of the type of MEA prepared, the cell mounted was sealed with a pressure of 2.5 Nm torque. The use of 100 μm thick gaskets achieved around 50 % of the MEA compression (15 % compression was achieved with 200 μm thick gaskets, see later). It is worth noting that all the assembly of materials was carried out under N_2_ atmosphere to prevent carbonation. Subsequently, the assembled cell was connected to the lab-built fuel cell bench after ensuring that there was no air in the fuel cell bench system.

#### 2.3.4 Fuel cell experiments

Once the MEA was prepared, it was tested on a lab-built fuel cell test bench to evaluate its performance via electrochemical characterizations, such as (i) polarization curve and if possible (ii) cyclic voltammetry to evaluate the electrochemical active surface area (ECSA) of the catalyst.

Prior to any test, fully humidified hydrogen and nitrogen (2 NL h^−1^ = 33.3 SCCM) were introduced to the anode and cathode sides, respectively, to prevent MEA drying during the gradual increase of the cell temperature to 60 °C. Once the desired temperature was reached, fully humidified nitrogen was replaced at the cathode by oxygen at 95 % RH and then the OCV was recorded. Subsequently, the activation step (conditioning or cell break-in) was performed with a flow rate of 12 NL h^−1^ (=200 SCCM) at both electrodes, at 95 % RH and 60 °C.

#### 2.3.5 Activation step procedure

The current was gradually increased every 5 min with a step of 20 mA, from 0 An until reaching the current corresponding to a voltage around 0.3 V as the voltage limit. Once the current – voltage (CV) curve has been collected, the maximum current value can be set. We define the half-current value as half of the maximum current value obtained in the CV curve. After the activation step, the cell voltage stabilized during 1 h at the half-current value. During this stage, the working conditions remain unchanged, that is, relative humidity 95 % on both electrodes, cell temperature at 60 °C, and flow rate at 12 NL h^−1^ for both hydrogen and oxygen gases.

#### 2.3.6 Polarization curves

The polarization curves were collected under the same conditions as during the break-in, except the flow rate at both electrodes. In fact, 2 flow rates were tested here, one at 12 NL h^−1^ and the other at 24 NL h^−1^ (see Table S1 for conversion of gas flow to stoichiometry). Except for further precision, each polarization curve resulted in an average of two measurements of voltage versus current. The first measurement started at 0 A (OCV) and the current gradually increased to 50 mA until the measured voltage reached 0.3 V. The second measurement started at the last point of the first measurement. The current was progressively decreased until it reached 0 A. Each current step took 2 min, and the average of the measured voltage was calculated.

#### 2.3.7 Cyclic voltammetry

Cyclic voltammetry tests were performed to evaluate the ECSA of the prepared MEA sample. First, fully humidified N_2_ was filled at both electrodes at 2 NL h^−1^ for 15 min. The fuel cell was operated using the classical cell (see later), with a flow rate of H_2_ at 2 NL h^−1^ on the anode side, while N_2_ is reduced to 0 NL h^−1^ on the cathode. The voltage sweep rate was 50 mV s^−1^ and the voltage range was between 0.04 and 0.8 V. The mean hydrogen adsorption/desorption charge, *Q*_*H*_ was determined in Eq. (3), by integrating the two hydrogen adsorption/desorption peaks across the obtained cyclic voltammetry curves.

Subsequently, the ECSA was calculated by Eq. (4).

(3) $$Q_{H}=\frac{\left(Q_{ox}-Q_{red}\right)}{2}$$

(4) $$ECSA=\frac{Q_{H}}{C_{pt}.\;L_{pt}}=\lfloor \frac{m^{2}}{g_{pt}}\rfloor$$

where Q_H_ (in C cm^−2^) is the charge density obtained from cyclic voltammetry curve, C_pt_ (=210 μC cm^−2^_Pt_) is the charge associated with the formation of a monolayer of adsorbed hydrogen on Pt, and L_pt_ is the platinum load in mg cm^−2^.

#### 2.3.8 Water management study

The water management was studied using two kind of cells, but the active area remains unchanged (7.22 cm^2^): (i) the classical cell and (ii) the reference electrode cell (a cell with a reference electrode inside the cell) is the lab-built fuel cell equipped with a hydrogen-fed reference electrode to measure the anode potential [38]. Contrary to the “classical” cell where a global voltage at both electrodes is measured for a given current, here we can separately measure the anode voltage (U_a_) and the global voltage (U). The cathode voltage (U_c_) is then calculated via Eq. (5).

(5) $$U_{c}=U-U_{a}$$

where U_c_, U and U_a_ are the voltage of the cathode, cell and anode, respectively.

The water management was investigated (i) with a classical cell by varying either flow rates at the both electrodes (12/12 or 24/24 NL h^−1^) at 95 % of RH or dew points of the gases and (ii) with the “reference electrode” cell. The cell temperature remains unchanged and is maintained at 60 °C. For each variation of parameter, a polarization curve was collected. The temperature of the cell and both electrodes was expressed in the following format: anode/cell/cathode.

For clarity, the operating conditions with the classical cell were.

1) The flow rate of the both electrodes were set first at 12/12 and after at 24/24 NL h^−1^; the temperature of the cell and RH set at 60 °C and 95 %, respectively.
2) 4 dew points were tested at the anode side (59, 58, 57 and 54 °C), whereas the dew point at the cathode side was kept at 60 °C. The temperature of the cell was also kept at 60 °C. The flow rate was set at 12/12 NL h^−1^.

With the reference electrode, the experiments were carried out at 60 °C, 95 % RH, but with different gasket thicknesses (100 and 200 μm) and ink preparations.

Each MEA was tested until holes appeared (MEA death, *i.e.,* a sudden drop in voltage to 0 V). As a result, some experiments do not reach the end of the scheduled tests due to materials degradation.

## 3 Results and discussion

### 3.1 Morphologies of catalyst layers (CLs) prepared with different ionomer contents

**Fig. 1:**
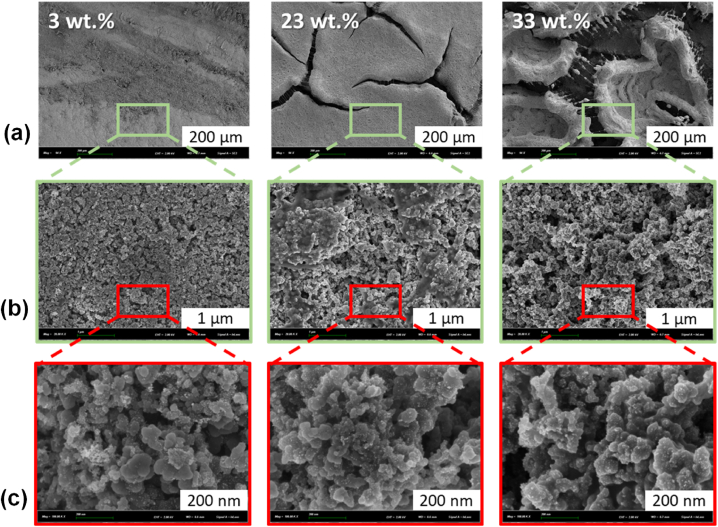
SEM images of the prepared GDEs samples with different ionomer contents at 3, 23 and 33 wt% (GDE-3%, GDE-23 % and GDE-33 %), images with magnification (a) 94× (scale bars 200 μm), (b) 20.000 x (scale bars 1 μm) and (c) 150.000 x (scale bars 200 nm).

The catalyst layers prepared with different ionomer contents were visualized by SEM images and measured by a tactile profilometer. Fig. 1 shows the surface morphology of GDEs prepared with different ionomer content at 3, 23 and 33 wt% at different magnifications ((a) 200 μm, (b) 1 μm and (c) 200 nm). At the scale of 200 μm, it seems that the cracks are more important and deeper when the ionomer content increases. Interestingly, the presence of cracks is not visually observed on the surface of CCM samples prepared with the same inks, especially with the ink containing 33 % of ionomer content. The results shown in Fig. 1 can be explained by a difference of hydrophobicity between the two supports, *i.e.*, the MPL and the membrane. The hydrophobic character of the MPL promotes the formation of a distinct and isolated block of electrodes (Pt/C + ionomer) that are randomly distributed on the surface of the GDE. This is especially true since the ionomer contents in the catalyst inks are high. With this kind of morphology, it seems that the prepared samples appear to be more porous due to the paths generated by the cracks. On the contrary, no cracks are detected and a denser and more compact electrode on the surface of the CCM-3% is observed as shown in Fig. 1 (a-c, 3 wt% of ionomer).

**Fig. 2:**
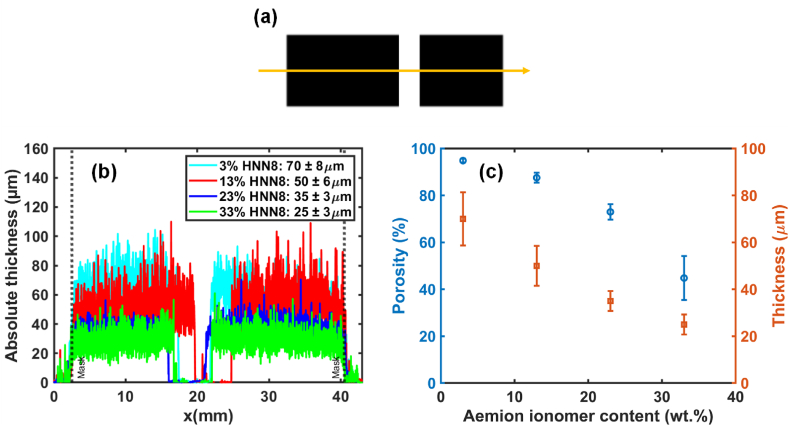
(a) The particular shape of the samples for profilometry, the yellow arrow indicates the direction of the measurements, (b) the thickness of the deposited layers loaded at 1 mg cm^−2^ of Pt, prepared with different ionomer contents (%) in the catalyst inks: 3 in yellow, 13 in red, 23 in blue, and 33 in green, (c) porosity calculated from the thickness measured in (b) and reported in the same graph. (For interpretation of the references to colour in this figure legend, the reader is referred to the Web version of this article.)

Fig. 2(a) shows the particular shape of the sample prepared for profilometry measurement for each prepared ink. Following the yellow line, the thickness of the coating layers was measured, and we can also check the homogeneity of the coating. Fig. 2(b) shows the thickness of the coated samples with different amounts of ionomer measured by the tactile profilometer. As shown, the more ionomers the ink contains, the thinner the deposited layer. This behavior has already been observed in our previous work when studying catalytic inks for PEMFC by varying the amount of Nafion ionomer [39]. In fact, ionomer not only transports the wished ions, but also plays as binder. During coating, evaporation of the catalyst ink solvent promotes gluing of the platinum and carbon particles with the ionomer. The higher the ionomer content in the catalyst ink, the greater the amount of ionomer covering the platinum and carbon particles, and the more the particles stick together. That is why increasing the ionomer content in the catalyst ink allows to decrease the porosity (voids between particles) of the prepared electrode and also decrease the distance between catalyst particles. Here, we have a gradual decrease of porosity according to the increase of anionic ionomer content (Fig. 2(c)), suggesting that the coverage of pores is proportional to the amount of Aemion® ionomer. With a 3 % Aemion® ionomer content, the amount of binder-ionomer is not enough to cover well the amount of catalyst particles (Pt and C), thus the resulting coating is very crumbly and can easily detach from the substrate. Therefore, this sample is not electrochemically characterized.

By crossing the SEM and profilometer results, it reveals that the sample prepared with 3 % of ionomer content is the thickest and most porous sample despite its dense surface appearance. On the contrary, the sample prepared with 33 % of ionomer content is the thinnest and densest electrode (near 50 % of porosity within 22 μm of CL). It should be mentioned that the characterized samples were in dry and iodide form. It is possible that the shape of the electrode could be changed in a different hydration or anionic environment. Indeed, the swelling of the Aemion® membrane is quite high in the hydroxide form (2.5 times higher in volume in 3 M KOH) so it could be that in the hydroxide form, the swelling could prevent or block partially the transport of gases, resulting in a decrease in the performance of AEMFC. To verify this eventuality, all the prepared samples were tested in AEMFC, except for the samples prepared with 3 % amount of ionomer for the reason mentioned above.

### 3.2 AEMFC's tests with CCM – based MEAs

**Fig. 3:**
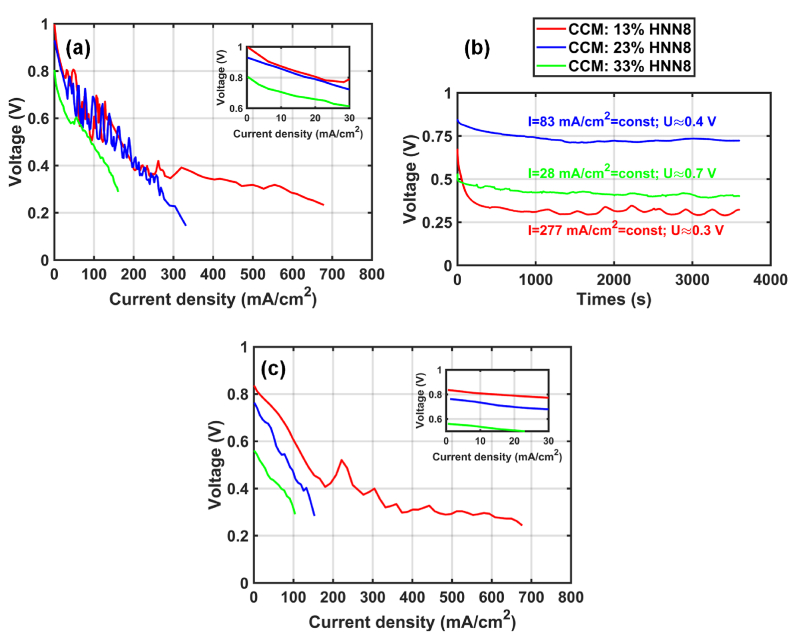
(a) Activation, (b) stabilization step and (c) polarization curves of the CCM–based MEAs with the 3 different ionomer contents (CCM-13 %, CCM-23 % and CCM-33 %) at 60 °C, 95 % RH.

Here we tested the 3 CCMs prepared with the 3 different ionomer contents (13, 23, and 33 wt%), respectively: CCM-13 %, CCM-23 % and CCM-33 %. Before any electrochemical test, each CCM sample was converted to hydroxide anion form. Then, it was assembled in the fuel cell and connected to the bench as described in Section 2.3.2. As soon as the fuel cell bench reached the set conditions, that is, T = 60 °C, 95 % RH, the OCV value (initial OCV in Table 2) was checked to avoid any problems with MEA construction or cell assembly, such as a hole or short-circuit, before launching the activation step. Fig. 3 gathers the OCV values, the CV curves of the break – in (activation step) and stabilization process of the 3 as-prepared CCMs. As it is pictured in the insert in Fig. 3 (a, or in the legend), the OCV value of the prepared CCMs seems to decrease with the increase of the ionomer content in the catalyst ink: 0.98 to 0.87 for the CCM-13 % to the CCM-33 %, respectively. During the activation step, the voltage fluctuates greatly with each increase in current density and the highest current density at 0.3 V is obtained with the CCM-13 % (Fig. 3(a)). After this step, the half-current was calculated and set to the stabilization step of each tested CCM (Fig. 3 (b)). From the latter, we can see that the potential remains rather stable at the stabilization step by imposing the half-current density (see section 2.4.1). However, the OCV of the 3 CCMs measured at the beginning of the stabilization step is lower than that of the activation step, regardless of the amount of ionomer within the CCMs (see later for the explanation).

**Table 2:** Evolution of the OCV value at each step of the electrochemical characterization for each tested CCM prepared with 13, 23 and 33 % of ionomer content, respectively.

OCV	Initial OCV	Activation step	Polarization step
CCM-13 %	0.99	0.98	0.83
CCM-23 %	0.91	0.93	0.78
CCM-33 %	0.87	0.87	0.59

Then, the polarization curves were collected for each tested CCM and the average value was plotted in Fig. 3(c). As in the activation step, the behavior of the 3 tested CCMs is rather similar, except for the OCV value. The CCM-33 % gives the lowest performance, near 120 mA cm^−2^ at 0.3 V. In contrast, CCM-13 % demonstrates the highest performance with a maximum of current density at 600 mA cm^−2^ at 0.3 V. This value is rather low compared with that reported from the literature [10], where the sample of 0.96 cm^2^ was prepared with 0.45 mg_Pt_ cm^−2^ of Pt loading and 10 % of ionomer content [10]. Although our Pt loading is twice that of the literature, the low performance may also be due to the low gas flow rate used at both electrodes and the choice of GDL at the both electrodes. Indeed, to reach 1 A cm^−2^ at 0.3V, the authors [10] applied an assymetrical design with different (i) types of GDL and (ii) flow rate of 50/100 ml min^−1^ for H_2_/O_2_ respectively for a 0.96 cm^2^ active surface.

Regarding now the OCV value of each tested CCM, we can see that its decrease is even more severe than that of the previous two electrochemical characterization steps. For clarity, the OCV value at each step of the electrochemical characterization is summarized in Table 2. The latter highlights the severe drop of the OCV value at each electrochemical test, suggesting a possible degradation of the membrane and ionomer over time.

Several hypotheses about the degradation of the membrane and ionomer were formulated to explain (i) the difference in OCV as a function of the amount of ionomer in the CCM and (ii) the gradual decrease in OCV as a function of experimental time. The first assumption is the high concentration of KOH used during the anionic conversion step, as well as the heating temperature (80 °C) during the coating procedure. The second assumption is the solvent isopropanol used to prepare the inks, which can dissolve the membrane due to a low difference in solubility between the solvent and polymer [22]. The third and last assumptions are based on the mechanical stress of the prepared CCMs during the anion conversion step. The swelling of the electrode occurs before the swelling of the membrane when the CCM is immersed in potash because the water must pass through the electrodes before it reaches the membrane. It can produce during this hydration transient step a tensile stress on the membrane not yet swollen and stretched by the swollen electrodes.

Due to the specificities of the Aemion® membrane and ionomer given by the supplier [36,40,41], the first hypothesis seems to us very unlikely. Now, as regards the second assumption, there may not be enough difference in solubility between the membrane and the chosen solvent. As a result, the membrane may be weakened during the coating step due to a possible solubilization of membrane with the presence of isopropanol. This is a possible assumption, because of the rapid dissolution of the membrane that was soaked in isopropanol and then immersed in water during an ex-situ experiment (Fig. S1). However, all the prepared CCMs with a similar Pt loading should normally receive a similar amount of solvent during the coating step. If this assumption is correct, the OCV value should be similar for all the CCMs tested and not vary with the amount of ionomer. However, it is possible that the drying time differs depending on the ionomer loading because the ionomer-bound solvent is likely to dry less quickly than the carbon-bound solvent. Thus, a high ionomer loading would result in a longer contact between the membrane and the solvent.

Then, we move to the last assumption. If we assume that the membrane is made of 100 wt% of ionomer content and its thickness swelling is 1.6 times higher than in dry and in iodide form, we can then calculate the corresponding swelling that the electrode can undergo during the anion conversion step. As seen in Table 3, the higher the ionomer content in the catalyst layer, the more pronounced the deformation of the catalyst layer.

**Table 3:** Characterization of the CCMs prepared with different ionomer contents.

CCMs	1st OCV (V)	Thickness in dry and iodide form (μm)	Thickness swelling in OH^−^ form^a^
CCM-3%	–	70 ± 8	0.05
CCM-13 %	0.98	50 ± 6	0.2
CCM-23 %	0.93	35 ± 3	0.37
CCM-33 %	0.87	25 ± 3	0.53

a Assumption: If the ionomer content of the membrane is 100 % - the thickness of the membrane increases 1.6 times (measured in 3 M KOH).

As the swelling of the membrane is quite important, one can imagine that the swelling rate of the electrode can vary with its thickness and amount of ionomer. The thinner the electrode, the faster the swelling and the higher the deformation in the case of a high ionomer content. As a result, CCM-33 % with the thinnest electrodes and the highest amount of ionomer among the prepared CCM is more impacted by the difference of swelling between the electrodes and the membrane. When CCM-33 % has been immersed in a 3 M KOH solution, the electrodes first swell, but the part of the membrane sandwiched between the 2 electrodes remains dry, resulting in a severe transient tensile stress in the plane direction of the membrane. With thicker electrodes and a lower amount of ionomer, a similar phenomenon occurs inside the membrane, but with less intensity, due to a less rapid diffusion of the electrolytes and less deformation of the electrode. For this reason, the OCV value varies with the amount of ionomer in the CCMs.

Concerning now the degradation over time, several reasons can explain the progressive drop of the OCV value for each tested CCM. One reason may be the high flow rate used in the AEMFC testing, which may promote degradation of the already defective membrane. However, this is not the only reason for the decrease of OCV. In fact, we must also consider sorption hysteresis and water management as membrane degradation parameters. We know that the used Aemion® membrane has a sorption hysteresis, which can affect water transport when the relative humidity changes. At high current densities or currents, the AEMFC produces more water than at low currents. This means that at high currents, possible drying of the membrane and flooding of the catalyst layer can occur on the cathode and anode side, respectively. At low current, drying of the membrane on the anode side can occur. In both cases, a change in membrane hydration occurs, that may affect the organization of water molecules within the membrane due to the sorption hysteresis (see Fig. S2). During the desorption step, some of the water molecules do not surround the IESs (ion exchange sites) but are bound to polymer chains. Because IESs are not protected by many water molecules, they can undergo degradation and/or reduce the transport of hydroxide anions, depending on the relative humidity level in the medium.

Of course, a post-mortem analysis would be useful to confirm all the hypotheses formulated in this work to explain the decrease in OCV observed with the prepared CCM-based MEAs. However, all the samples tested ended prematurely with a hole, so post-mortem analysis could not be carried out in our case. According to the literature [12], a post-mortem study of CCM manufactured with Aemion® membrane was performed after 12 h’ testing in H_2_/O_2_ mode, at 70 °C and 90 % RH. The results showed an excessive swelling and dimensional instability of the membrane, with an increase of the area specific resistance (decrease of the ion exchange capacity), suggesting the membrane degradation.

Since the prepared CCMs are subjected to transient tensile stresses during the anionic conversion step, we now turn to GDE-based MEAs.

### 3.3 AEMFC's tests with GDE – based Meas

**Fig. 4:**
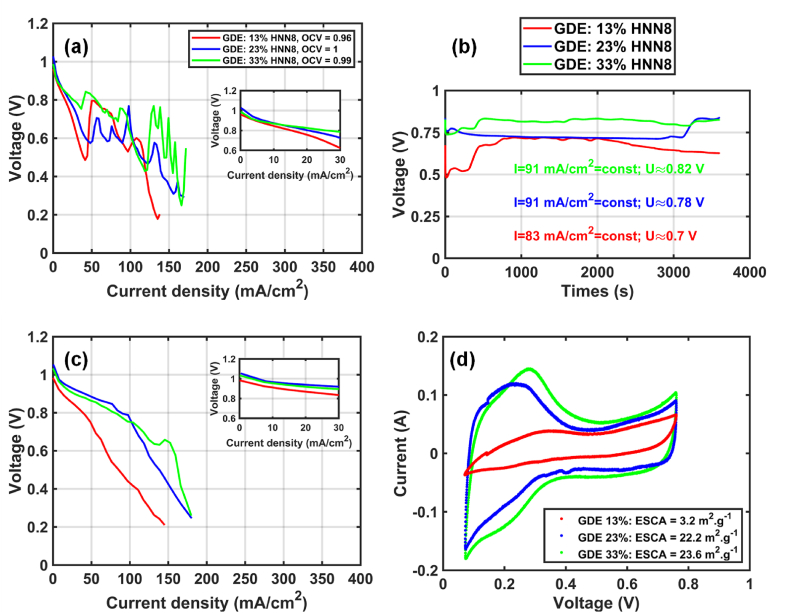
*(a) Activation, (b) stabilization step, (c) polarization curves and (d) cyclic voltammetry measurement result (legend) of the GDE-based MEAs with the 3 different ionomer contents (*GDE-13 %, GDE-23 % and GDE-33 %) at 60 °C, 95 % RH.

Here, we tested the 3 GDE-based MEAs that were prepared with the 3 catalyst inks containing different amounts of ionomer (13, 23 and 33 wt%): GDE-13 %, GDE-23 % and GDE-33 %. The anion conversion step, the electrode and membrane assembly, as well as fuel cell connection, were performed as described above. Fig. 4 illustrates the OCV values and CV curves of break-in and stabilization process of the three GDE-based MEAs in the fuel cell bench. At first glance, the OCV of the 3 GDE-based MEAs tested remains similar regardless of the amount of ionomer used in the catalyst inks. This result confirms us that there is no damage in the GDEs and the membrane during the anion conversion step. As for the CCMs activation process, the cell potential of the 3 tested GDE-based MEAs noticeably fluctuates over the current density range (Fig. 4(a)) but remains rather constant during the stabilization step at the half-current (Fig. 4(b)). Unlike the CCMs’ activation step, the 3 tested GDE-samples seem to behave similarly. Nevertheless, the potential of the GDE-13 % is a little bit lower than the other 2 GDE-samples, due to a poor contact between membrane and GDE (see below).

Turning now to the polarization curves of the 3 prepared GDE-based MEAs, Fig. 4(c) illustrates the average of the 2 polarizations curves (Table S2,see Supported Information). At first glance, the OCV value is rather constant for the 3 samples studied, suggesting here that there is no degradation or that the degradation rate is very slow compared to the CCM-samples. Furthermore, there is a change in the order of the performance in Fig. 4(c). The higher the ionomer content in the GDE, the better the performance in the AEMFC. This order seems to follow the thickness of the deposited catalyst layer and the covering of ionomer on catalyst particles. The thicker the electrode, the worse the gas and water transport. But without sufficient covering of ionomer on Pt/C, the triple-phase boundary is poor due to a low ionic conductivity and water and gas transport.

Regardless of the OCV value, the performance obtained with the GDE-samples is rather low in comparison with CCM-samples in Fig. 3(c). This result can be explained by a weaker interface contact between the GDE and the membrane, which was previously reported in the literature regarding GDE-based MEAs vs. CCM-based MEAs in PEMFC [42,43]. Despite poorer interfacial contact between GDEs and the membrane, the insufficient amount of ionomer binder also affects this interfacial contact, especially in the case of GDE-13 % (Fig. S3, see supported information). This problem was revealed after the MEA was disassembled at the end of the fuel cell test, where the GDEs are not stuck together with the membrane surfaces, although the same gaskets and sealed pressure (2.5 Nm torque) were used for all prepared MEAs. With the other 2 samples at higher ionomer contents, there is a good adhesion between GDEs and membrane surface. In addition, the insufficient amount of ionomer in the GDE-13 % is also evidenced by the ECSA measurement of the tested GDE-samples in Fig. 4(d). Indeed, the ECSA of the GDE-13 % is around 3 m^2^ g^−1^, whereas the other samples are near 22–23 m^2^ g^−1^.

10.13039/100014337Furthermore, the polarization curves (by increasing and decreasing current) are also sensitive to the lack of ionomer amount in the case of the GDE-13 % (Table S1, see Supported Information). Except for the latter, the other 2 samples show a better polarization curve in the 2nd measurement (by decreasing current) and the issue of the sorption hysteresis and gas transport can be observed at high current density part (>50 mA cm^−2^, Table S1, see Supported Information). Although the sorption hysteresis cannot be avoided, water and gas transport may be optimized. That's why we did an attempt for studying the water management in AEMFC.

### 3.4 Water management study

Several approaches have been tested to study the water transport in an operating AEMFC, such as (i) flow rate of gases, (ii) relative humidity of gases, and (iii) use of cell with a reference electrode inside the cell (this cell is called a reference electrode cell, with a hydrogen-fed reference electrode).

**Fig. 5:**
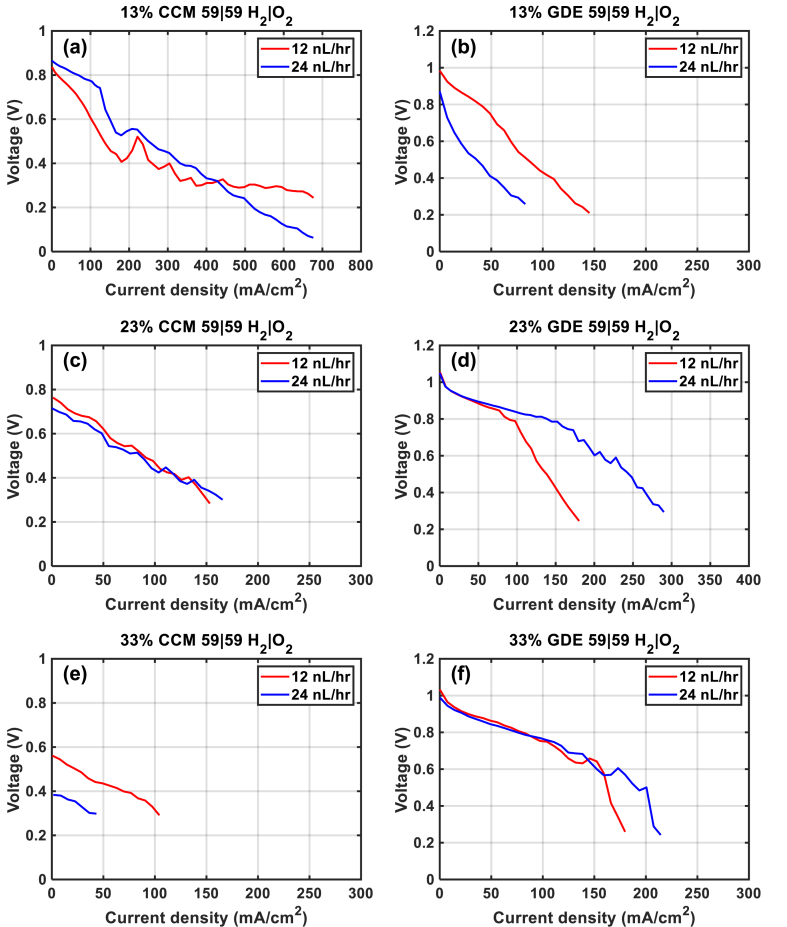
Polarization curves of CCM- (left) and GDE (right) -based MEAs (Pt loading at 1 mg cm^−2^) at 12 and 24 NL h^−1^.

(i) was tested with the CCM- and GDE-based MEAs prepared with the dispersion step (use of Ultra-Turrax) by increasing the flow rate from 12 to 24 NL h^−1^, as shown in Fig. 5. Concerning CCM-based MEAs (Fig. 5 (a, c, e)), increasing the flow rate at both electrodes has not increased the performance of the samples in AEMFC. As seen in section 3.2 ((with a flow rate of 12 NL h^−1^), it could be that the defects initiated during the anionic conversion step may be the reason.

On the contrary, increasing the flow rate at both electrodes favor better AEMFC performance in the case of GDE-based MEAs (Fig. 5 (b, d, f)), except for GDE-13 % (Fig. 5 (b)), where a poor contact between the layers of the assembly was observed. In the case of GDE-23 %, the increase of flow rate can force the gas to diffuse into the electrodes and allow better water and gas transport, resulting in an almost doubling of the current density at 0.3 V. In the case of the GDE-33 % (Fig. 5 (f)), the increase in flow rate does not affect the performance in AEMFC so much, because of the rather thin thickness of the electrodes (near 25 μm, Fig. 2 (b)) and of the high ionomer amount within the electrodes. If the ionomer behaves like the membrane, the swelling of the ionomer inside of the electrodes may partially prevent the passage of gas.

**Fig. 6:**
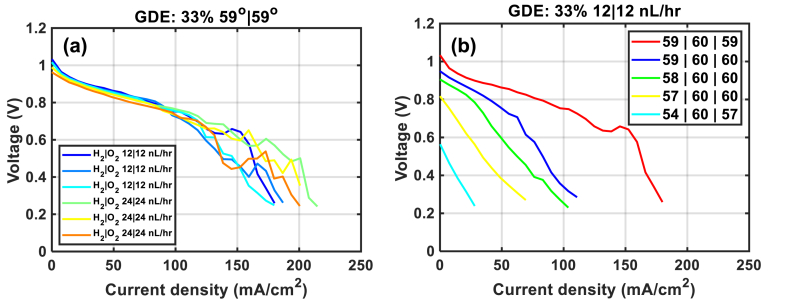
Water management with the GDE-33 % with (a) different debit at constant relative humidity (95 %) and (b) different relative humidity at 12 NL h^−1^ at both electrodes.

As the increase in flow rate does not impact too much the performance of the GDE-33 %, we tried to vary the dew points of the gases during the fuel cell tests (Fig. 6 (b)). The experiments were performed after the series of polarization curves with different flow rates (Fig. 6(a)). We underlined that the collection of each polarization curve took approximately 2 h (see section 2.4.2.)and among all the tested samples, only the GDE-33 % was the most stable in terms of performance (Fig. 6(a)) under the set operating conditions. However, a small drop of OCV can be remarked at the end of the series of polarization curves in Fig. 6(a). Note that the chronological order of the CV curves is given in order from the blue curve to the orange curve in Fig. 6(a) and from red curve to cyan curve in Fig. 6(b). When the dew point at the anode or cathode is varied by only a few °C, the performance is not reversible and there is a severe drop in the OCV value, resulting of a hole at the end of the experiments (visually observed after disassembly the cell). We believed that flooding and/or drying occurs at both electrodes. The loss of performance is probably due more to aging (probably due to the dimensional instability of the membrane) than to the change in humidity of the gases.

**Fig. 7:**
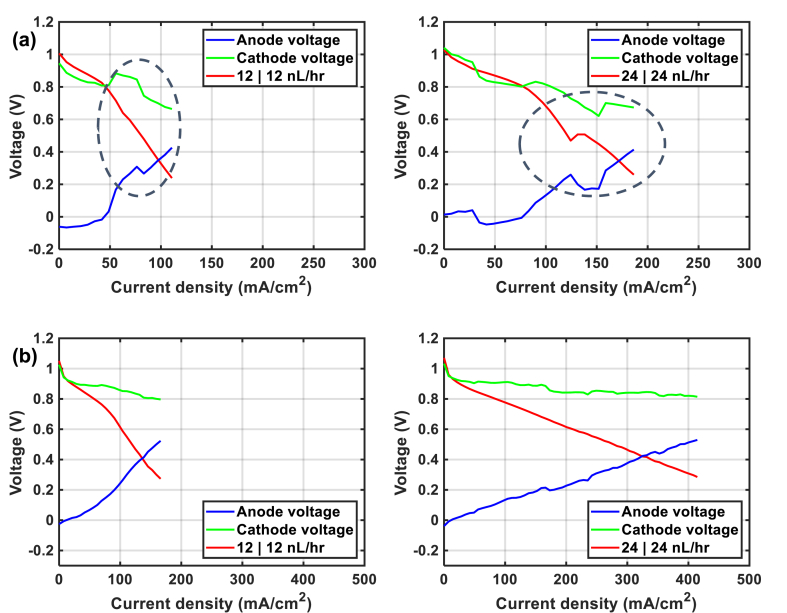
Polarization curves with the reference electrode in the cell measured at 12/12 (left) and 24/24 nL h−1(right) and mounted with (a) GDE-23%-unmixed (with a non-mixed ink) and 100 μm of Teflon gasket and (b) GDE-23 % with 200 μm Teflon gasket.

From the reported results, it seems to us that it is not easy to adjust the hydration level of the sample with 33 % of amount of ionomer once flooding or drying occurs. In order to understand what happens during fuel cell operation, we resorted to the use of the reference electrode cell (with hydrogen-fed reference electrode), which can help us to separate the voltage signature on the cathode side from that on the anode side. Here, we tested a GDE-23 % made with an ink prepared without ultra-Turrax step (called GDE-23%-unmixed) and a GDE-23 % with a 200 μm gasket. In fact, we would like to check on which parameter to play for a better water management: is it better to play with the electrode thickness or the gasket thickness. By decreasing the thickness by playing on the number of layers to reach 0.5 mg cm^−2^ of Pt loading (GDE-23%-0.5, Fig. S4), we can see a significant drop in electrode activity at low current density, as if there were no Pt in the CLs. For this reason, we preferred to change the ink preparation method to reduce the thickness of the CL. According to our previous work, the CL's thickness prepared without the step of Ultra-Turrax should be thinner than with the step of mixing step, in the case of Pt-Carbon Vulcan we have. Fig. 7 presents the performance and results that were obtained with the reference electrode cell at different flow rates of gases, at 95 % RH and 60 °C. Whatever the flow rate applied to the two electrodes, the voltages on the anode and cathode sides evolve similarly as a function of the current density, showing that there is a decorrelation of the signatures of the two electrodes. As previously, increasing the flow rate increases the global performance of the AEMFC (comparison of the red curves in Fig. 5).

**Fig. 8:**
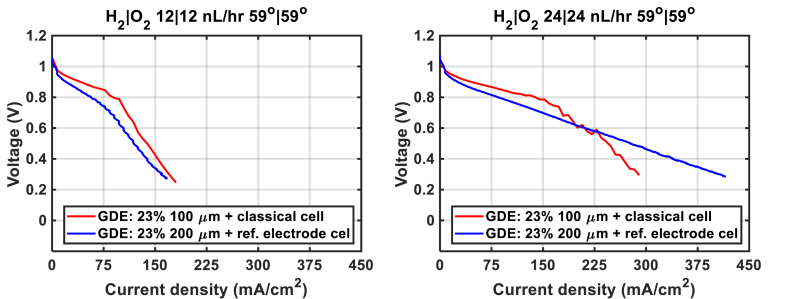
Polarization curve results obtained with 100 with 200 μm of gaskets with the reference electrode and the classical cell measured at 12/12 (left) and 24/24 nL h^−1^(right).

Regardless of the flow rates used and the MEAs, it seems that the beginning of the polarization curve on the cathode side (0–10 mA cm^−2^) is due solely to the overpotential for the oxygen reduction reaction. Overall, the decrease in voltage on the cathode side is less than on the anode side. The main problem comes from the anode side, where significant flooding is observed regardless of the MEAs and flow rates used, even at low current density. According to our results, a thinner electrode drowns easily (Fig. 7(a)), resulting in lower performance than a thicker electrode (Fig. 5(d)), especially when using 100 μm thin gaskets (50 % compression). In the case of a thicker gasket (Fig. 7(b)), the compression of the MEA is much lower (15 % compression), which should help a swollen MEA. At high velocity of gas, we can reach a higher current density (*i.e.*, >0.4 A cm^−2^) than the one with 100 μm gaskets reported in Fig. 5(d), near 0.3 A cm^−2^. From this experiment, it seems that we can improve the performance by increasing the gasket due to the change of MEA compression. However, when we compare the results obtained with 100 with 200 μm of gaskets (*i.e.*, Fig. 5 (d) vs. Fig. 7 (b) plotted in Fig. 8), we can observe that thicker gaskets do not improve performance in the test at 12 NL h^−1^, due to lower interface contact. With 24 NL h^−1^, we also observe lower interface contact when the amount of water produced is low (I < 225 mA cm^−2^), in other words when the MEA is not well swollen. When this current is reached, thicker gaskets promote the swelling of the MEA and delay the mass transfer limit, although flooding is still present. The results of this comparison show that MEA swelling is very high with both Aemion® membrane and ionomer, and that it is necessary to find a compromise between having a good contact interface or good water management when choosing the thickness of the gasket.

## 4 Conclusion

In this work, we attempted to optimize MEAs by varying the support, the amount of ionomer in the catalyst layers, as well as the water management to increase the performance of the AEMFC. For that, we prepared different CCM and GDE-based MEAs with different amounts of ionomer from 3 to 33 % in weight. The commercial ionomer and membrane used are Aemion® AP1–ENN8/HNN8 and Aemion® AF1–HNN8–25 respectively. The prepared samples were characterized morphologically by SEM and profilometer techniques and electrochemically by AEMFC polarization curves. The results of this study lead us to the following conclusions:

The study of ionomer content effect reveals that (i) higher the ionomer content, the denser and thinner the CL, and (ii) the morphology of the GDEs depends on the ionomer content, due to the difference in hydrophobicity between MPL and ionomer. The higher the amount of ionomer, the greater the segregation in the electrode. Regardless of the ionomer content within the prepared samples, the performance of CCM-based MEAs is higher than that of GDE-based MEAs, because of better interfacial contact. However, CCM-based MEAs have a lower OCV. The higher the ionomer content in the CCMs, the lower the OCV. This behavior is considered to be a consequence of a transient tensile stress during the anionic conversion step, as well as heating during evaporation of solvents. Although the alteration of a MEA can be caused by drying of the membrane and flooding of the electrodes, due to bad gas transport, the sorption hysteresis effect cannot be neglected in AEMFC with hydrocarbon membranes. This phenomenon can affect the polarization curves and change the distribution of water molecules within the polymer in a different way.

The study of water management reveals that.

(i) It is possible to increase the performance of rather thick GDE-based MEAs by increasing the flow rate;
(ii) Electrodes are very sensitive to changes in the dew point of gases, which leads to an irreversible decrease in performance;
(iii) The use of a reference electrode in the cell shows mainly the ORR at the cathode at low current densities and a flooding at the anode at high current densities, respectively.

In addition, increasing the thickness of the Teflon gasket can favor membrane and electrode swelling and thus stabilize water management, while reducing the interface contact of the low current density.

## Data availability statement

Data will be made available on request.

## CRediT authorship contribution statement

**Zarina Turtayeva:** Writing – original draft, Methodology, Investigation, Formal analysis, Data curation. **Feina Xu:** Writing – review & editing, Validation, Supervision, Methodology, Investigation, Data curation. **Jérôme Dillet:** Software, Methodology. **Kévin Mozet:** Software, Methodology. **Régis Peignier:** Visualization. **Alain Celzard:** Writing – review & editing, Visualization, Validation. **Gaël Maranzana:** Writing – review & editing, Validation, Supervision, Methodology.

## Declaration of competing interest

The authors declare that they have no known competing financial interests or personal relationships that could have appeared to influence the work reported in this paper.
