# Supplementary material for: Investigation of membranes-electrodes assemblies in anion exchange membrane fuel cells (AEMFCs): Influence of ionomer ratio in catalyst layers
Source: Heliyon. 2024 Apr 12;10(8):e29622. doi: 10.1016/j.heliyon.2024.e29622 (PMC11046124; doi:10.1016/j.heliyon.2024.e29622)
Supplement: Multimedia component 1 [file mmc1.docx]

**Supplementary information**


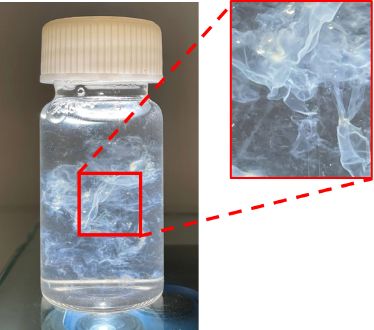


**Figure S1**. Degradation of a piece of the membrane Aemion inside of a bottle of water after being soaked in IPA (isopropanol)

**Figure S2.** Sorption curve of Aemion^®^ membrane in chloride form at 30°C.

*
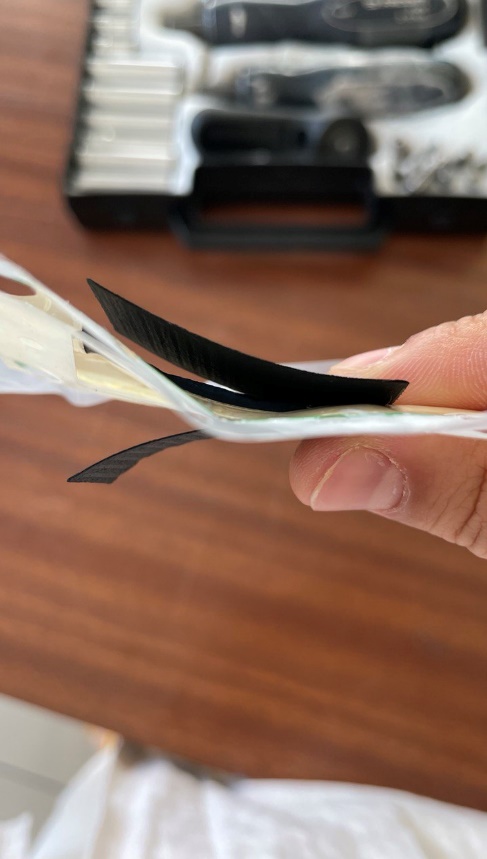
*

**Figure S3.** State of the MEA after cell disassembly showing the limited interfacial contact between 13 wt.% ionomer content-based GDE and membrane.

**Figure S4.** Polarization curves of GDE-23%-0.5 (Pt loading at 0.5 mg cm^-2^)

**Table S1.** Converting gas flow into stoichiometry

| Flow rate | | 12 NL h^-1^ (200 SCCM) | | 24 NL h^-1^ (400 SCCM) | |
| --- | --- | --- | --- | --- | --- |
| Current (A) | Current density (A cm^-2^) | S(H_2_) | S(O_2_) | S(H_2_) | S(O_2_) |
| 1 | 0.14 | 28.72 | 57.44 | 57.44 | 114.88 |
| 2 | 0.28 | 14.36 | 28.72 | 28.72 | 57.44 |
| 3 | 0.42 | 9.57 | 19.15 | 19.15 | 38.29 |
| 4 | 0.55 | 7.18 | 14.36 | 14.36 | 28.72 |
| 5 | 0.69 | 5.74 | 11.48 | 11.49 | 22.98 |

**Table S2.** Polarization curve measurements on increase (in blue)/decrease (in red) of current. The polarization curves shown in the paper were obtained by averaging the two curves (red and blue curves), except for the CCM-13%.

|  | CCM | GDE |
| --- | --- | --- |
| 13% |  |  |
| 23% |  |  |
| 33% |  |  |
